# Supplementary material for: Protection of Chickens against H9N2 Avian Influenza Isolates with a Live Vector Vaccine Expressing Influenza Hemagglutinin Gene Derived from Y280 Avian Influenza Virus
Source: Animals (Basel). 2024 Mar 12;14(6):872. doi: 10.3390/ani14060872 (PMC10967311; doi:10.3390/ani14060872)
Supplement: Supplementary file 1 [file animals-14-00872-s001.zip › animals-2894620-supplementary.pdf]

# Supplementary Materials

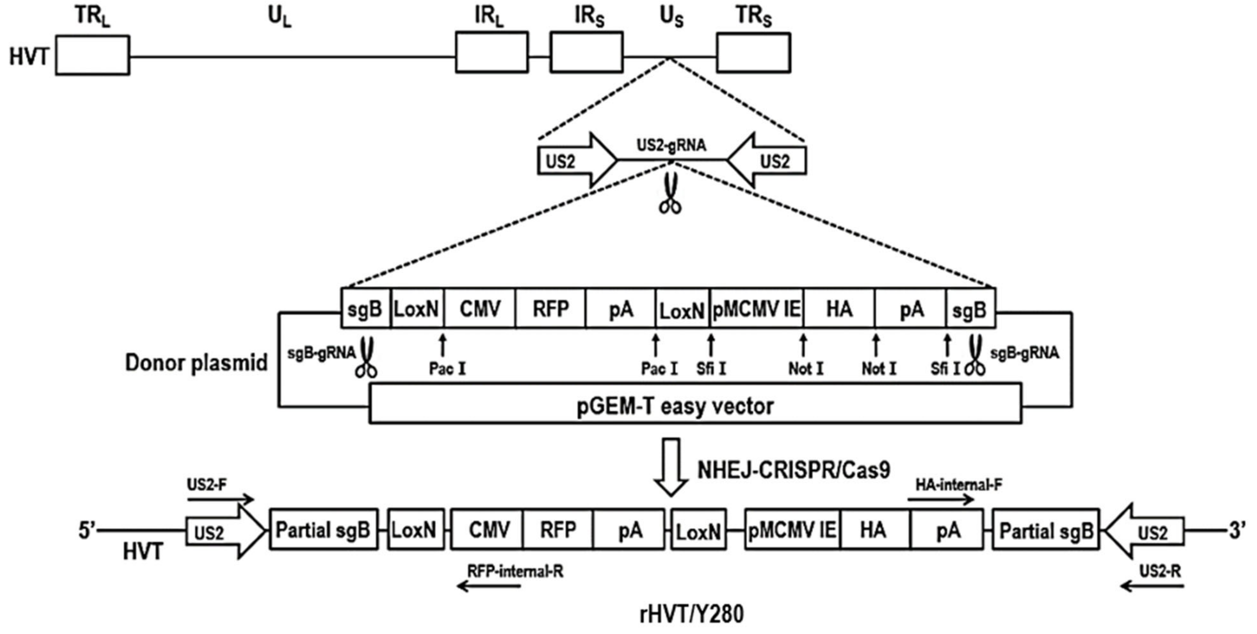


**Figure S1 |** Generation strategy for rHVT/Y280. The RFP expression cassette, surrounded by LoxN sites, is followed by the H9N2/Y280-HA expression cassette, both of which are flanked by sgB target sites.

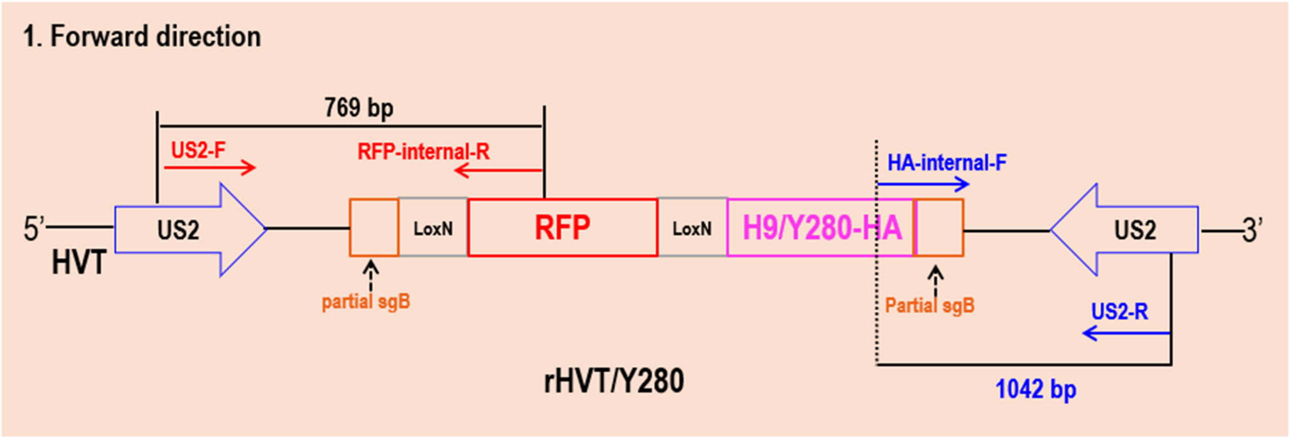


**Figure S2 |** PCR-based identification of rHVT/Y280 with RFP-H9/Y280 forward insertion. The primers US2-F/RFP-internal-R and HA-internal-F/US2-R were used, targeting bands of 769 bp and 1042 bp, respectively.

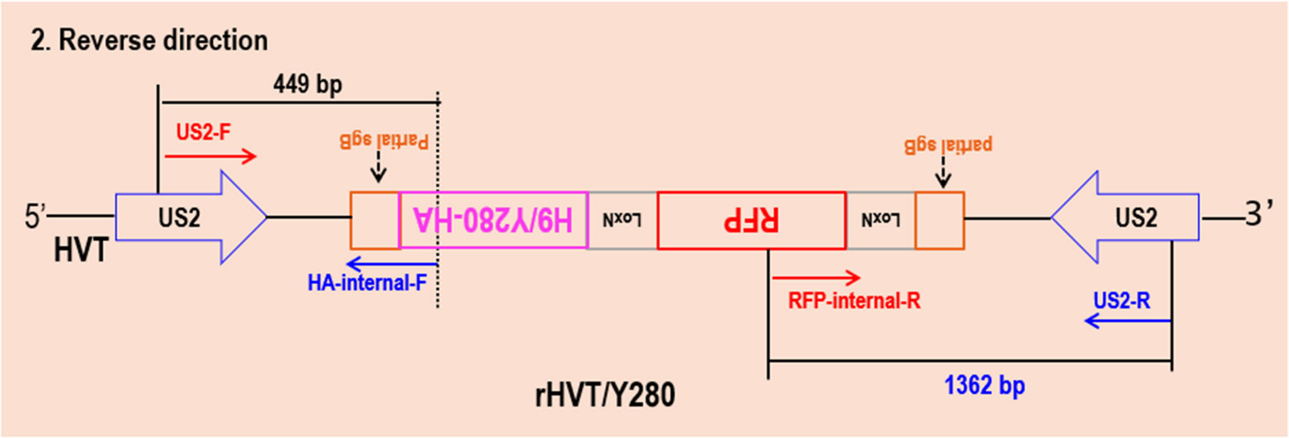


**Figure S3 |** PCR-based identification of rHVT/Y280 with RFP-H9/Y280 reverse insertion. The primers US2-F/HA-internal-F and RFP-internal-R/US2-R were used, targeting bands of 449 bp and 1362 bp, respectively.

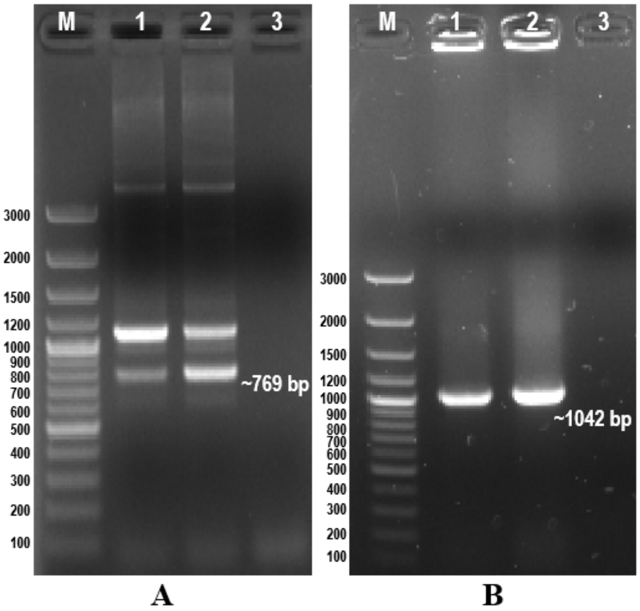


**Figure S4 |** Identification of rHVT/Y280 by PCR. (A) Identification of rHVT/Y280 using primers US2-F/RFP-internal-R. (B) Identification of rHVT/Y280 using primers HA-internal-F/US2-R.

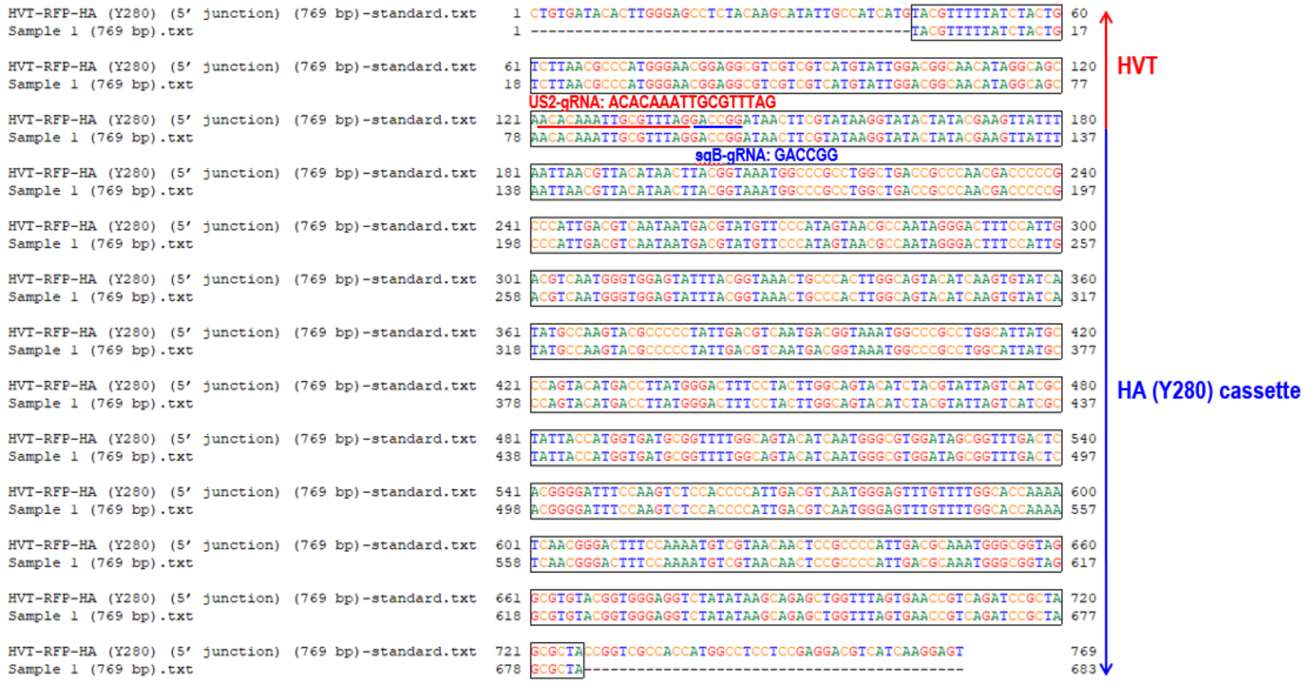


**Figure S5 |** Sequencing result of rHVT/Y280 with RFP-H9/Y280 insertion in the forward orientation using primer US2-F.

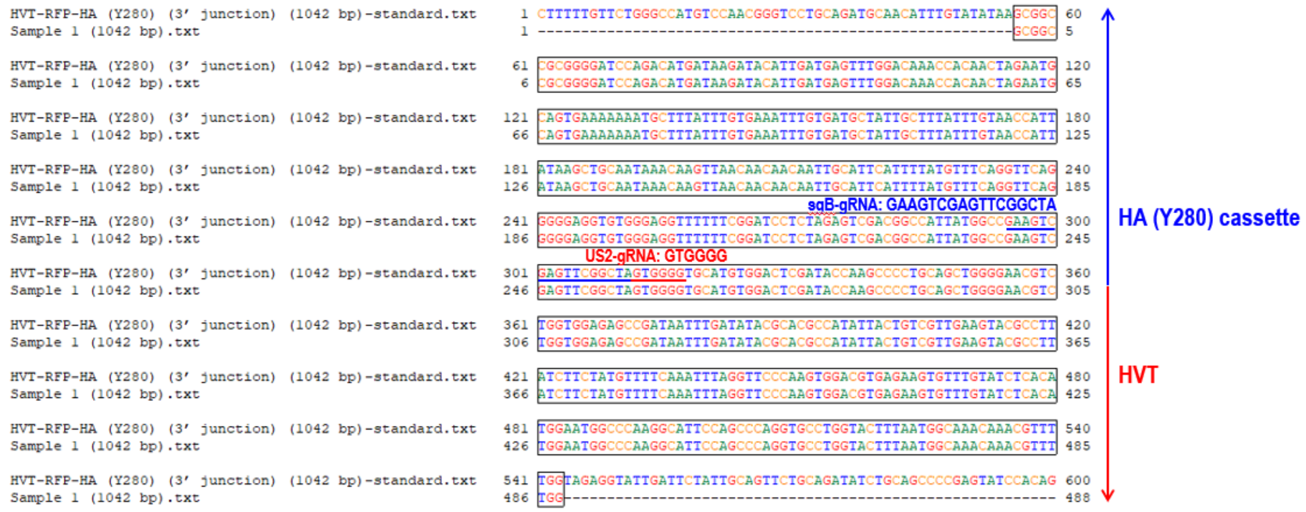


**Figure S6 |** Sequencing result of rHVT/Y280 with RFP-H9/Y280 insertion in the forward orientation using primer US2-R.

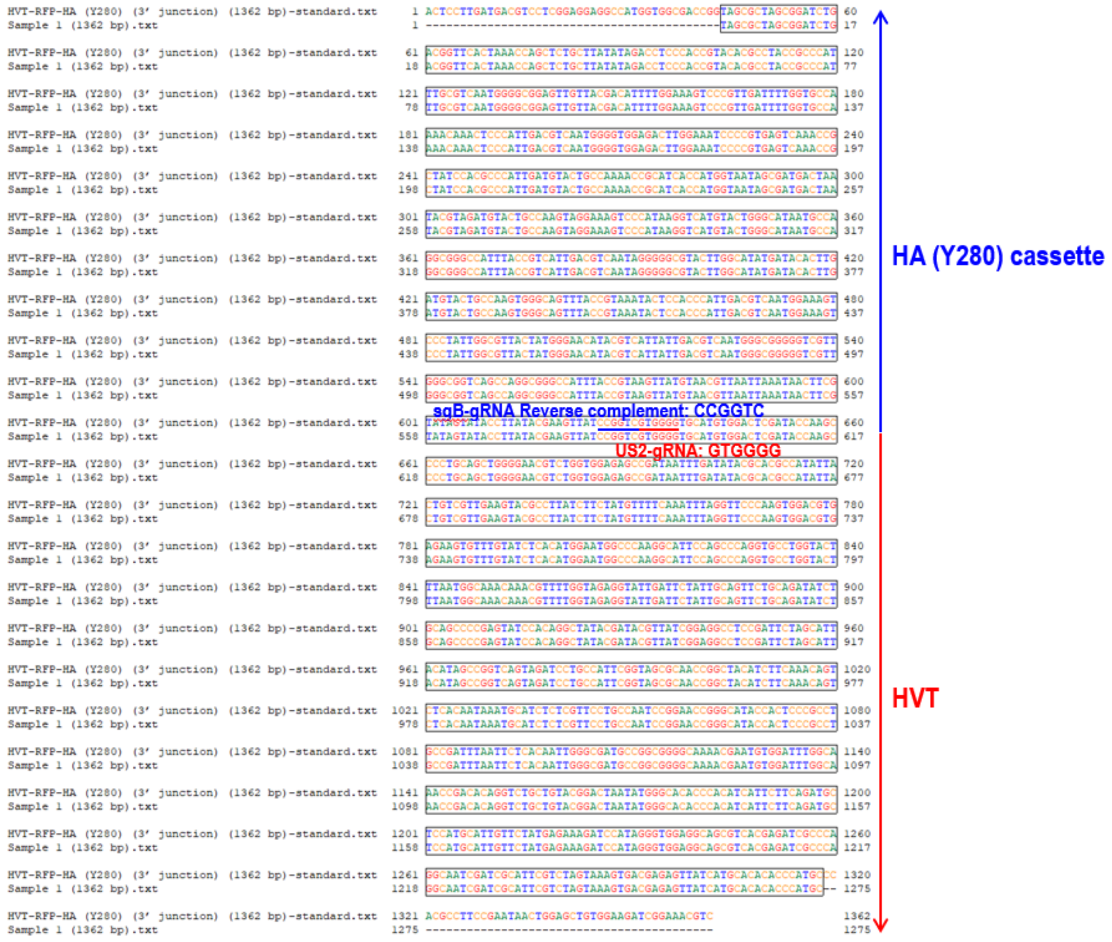


**Figure S7 |** Sequencing result of rHVT/Y280 with RFP-H9/Y280 insertion in the reverse orientation using primer US2-F.

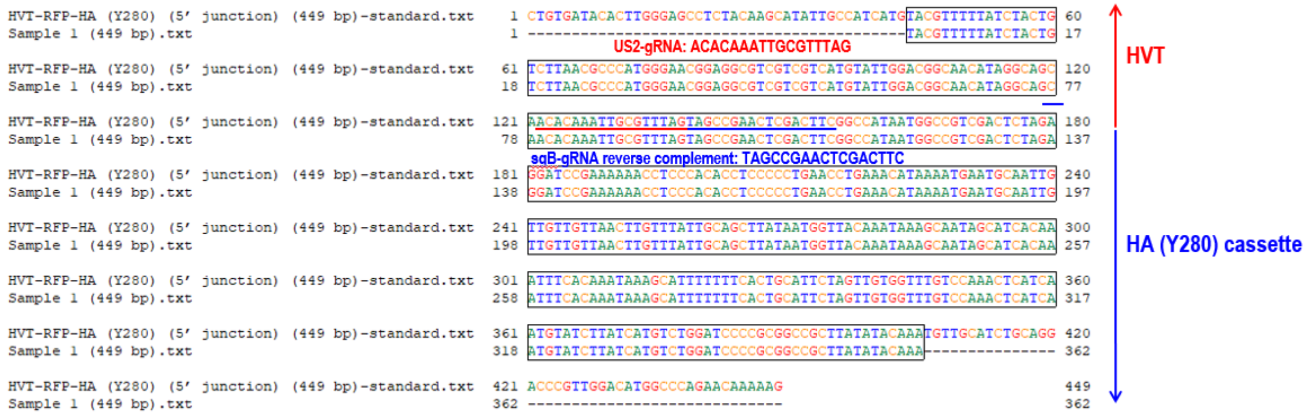


**Figure S8 |** Sequencing result of rHVT/Y280 with RFP-H9/Y280 insertion in the reverse orientation using primer US2-R.

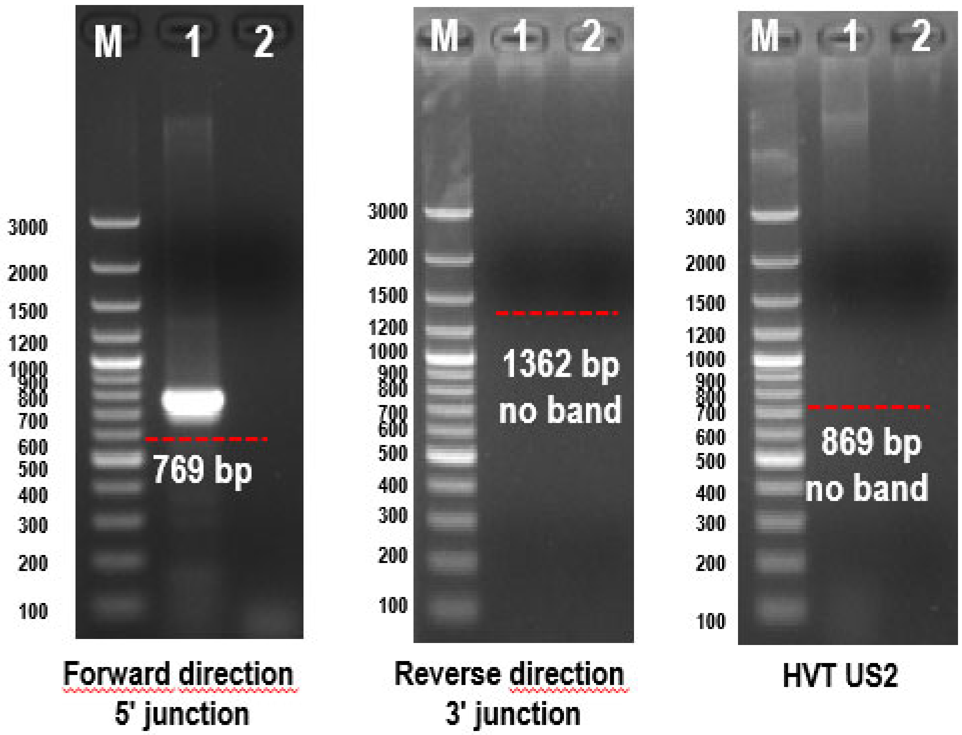


**Figure S9 |** Identification of purified rHVT/Y280 by PCR. Primers US2-F and RFP-internal-R were used for identifying the virus in the forward orientation. Primers RFP-internal-R and US2-R were used for identifying the virus in the reverse orientation. Primers US2-F and US2-R were used to detect the presence or absence of parental HVT virus.

**Table S1 |** List of primers used in this study

| Primer | Sequence (5'-3') |
|---|---|
| US2-gRNA-F | CACCACACAAATTGCGTTTAGGTG |
| US2-gRNA-R | AAACCACCTAAACGCAATTTGTGT |
| sgB-gRNA-F | CACCGAAGTCGAGTTCGGCTAGAC |
| sgB-gRNA-R | AAACGTCTAGCCGAACTCGACTTC |
| U6 forward | GACTATCATATGCTTACCGT |
| US2-F | CTGTGATACACTTGGGAGCC |
| RFP-internal-R | ACTCCTTGATGACGTCCTCG |
| HA-internal-F | CTTTTTGTTCTGGGCCATGT |
| US2-R | GACGTTTCCGATCTTCCACA |

**Table S2 |** Gross lesions against H9N2/Y280 infection in vaccinated chickens

| Group | Vaccination | Gross lesions (5 dpi) | | | |
|---|---|---|---|---|---|
| | | Trachea | Thymus | Kidney | Bursa |
| A | rHVT/Y280 | 0/12 (0%) | 0/12 (0%) | 0/12 (0%) | 0/12 (0%) |
| B | Inactivated H9N2/Y439 | 0/8 (0%) | 0/8 (0%) | 0/8 (0%) | 0/8 (0%) |
| C | Inactivated H9N2/Y280 | 4/8 (50%) | 4/8 (50%) | 3/8 (37.5%) | 0/8 (0%) |
| PC | PBS | 6/10 (60%) | 6/10 (60%) | 6/10 (60%) | 3/10 (30%) |
| NC | PBS | 0/6 (0%) | 0/6 (0%) | 0/6 (0%) | 0/6 (0%) |
